# Predictors of Retention among Men Attending STI Clinics in HIV Prevention Programs and Research: A Case Control Study in Pune, India

**DOI:** 10.1371/journal.pone.0017448

**Published:** 2011-03-11

**Authors:** Seema Sahay, Nikhil Gupte, Radhika G. Brahme, Amit Nirmalkar, Shilpa Bembalkar, Robert C. Bollinger, Sanjay Mehendale

**Affiliations:** 1 Division of Social and Behavioral Research, National AIDS Research Institute, Pune, India; 2 Clinical Trial Unit, Byramjee Jeejeebhoy Medical College, Pune, India; 3 Division of Epidemiology and Biostatistics, National AIDS Research Institute, Pune, India; 4 Johns Hopkins Center for Clinical Global Health Education, Johns Hopkins University, Baltimore, United States of America; 5 National Institute of Epidemiology, Chennai, India; University of Toronto, Canada

## Abstract

**Background:**

Retention is critical in HIV prevention programs and clinical research. We studied retention in the three modeled scenarios of primary prevention programs, cohort studies and clinical trials to identify predictors of retention.

**Methodology/Principal Findings:**

Men attending Sexually Transmitted Infection (STI) clinics (n = 10, 801) were followed in a cohort study spanning over a ten year period (1993–2002) in Pune, India. Using pre-set definitions, cases with optimal retention in prevention program (n = 1286), cohort study (n = 940) and clinical trial (n = 896) were identified from this cohort. Equal number of controls matched for age and period of enrollment were selected. A case control analysis using conditional logistic regression was performed.

Being employed was a predictor of lower retention in all the three modeled scenarios. Presence of genital ulcer disease (GUD), history of commercial sex work and living away from the family were predictors of lower retention in primary prevention, cohort study and clinical trial models respectively. Alcohol consumption predicted lower retention in cohort study and clinical trial models. Married monogamous men were less likely to be retained in the primary prevention and cohort study models.

**Conclusions/Significance:**

Predicting potential drop-outs among the beneficiaries or research participants at entry point in the prevention programs and research respectively is possible. Suitable interventions might help in optimizing retention. Customized counseling to prepare the clients properly may help in their retention.

## Introduction

With an estimated 2.3 million HIV infected persons, India has the third largest HIV burden in any country in the world [1]. One of the goals of the current third phase of National AIDS Control Program (NACP-III) in India is to halt and reverse the HIV epidemic by 2012 by implementing an integrated strategy focusing on prevention, care and treatment of HIV/ AIDS [2]. This goal can be achieved by maintaining the primary prevention continuum, effectively tracking the HIV incidence in various sub-populations and implementing appropriately evaluated prevention and therapeutic interventions.

Projections for the year 2031 marking 50 years of AIDS pandemic have indicated that almost three times the current resources will be required to control the epidemic by focusing on high impact tools, efforts to attain behavior-change and efficient and effective treatment [3]. All such efforts would require high level of utilization of services and programs by the stakeholders and their continued participation in the program. Retention in prevention programs, cohort studies and clinical trials is very critical and yet can be very challenging. The losses to follow-up (LTFU) might result from participants' loss of interest, inadequate oversight by the study investigators or absence of built-in mechanisms for tracking the study participants being lost [4]. Recent studies have shown that in resource poor countries, investigators can achieve high retention rates over long follow-up period in marginalized or “hard to reach” populations by employing special efforts which are expensive and management intensive [4], [5]. Health program managers and research scientists have to take necessary steps to ensure that their clients return to the health facility at the assigned time points. Hence, understanding of dynamics of retention of clients is likely to help in planning measures to retain people in prevention programs and research settings requiring long follow-up such as cohort studies and clinical trials. Our long-term prospective study provided an opportunity to estimate levels of retention and their predictors using a modeling approach in the context of various HIV prevention and research program related scenarios such as those described below. We present three possible scenarios in the area of HIV prevention and research wherein retention is crucial:

1. Primary prevention through Voluntary Counseling and Testing (VCT): We hypothesized that high uptake of voluntary counseling and testing services for HIV, an important primary prevention strategy of the National AIDS Control Program of India, would contribute to reliable estimation of HIV burden in various sub-populations and may guide in deciding strategies for secondary prevention and control of AIDS.
2. Cohort study: We hypothesized that individuals with high risk behavior who are retained in cohorts for longer durations provide opportunities for researchers to determine the incidence of HIV infection. This information can be used for monitoring the success of the program effectiveness or identify the need for new interventions.
3. Clinical trials: Our hypothesis was that individuals retained at precise multiple follow-up time points in clinical trials can help the program managers identify effective interventions for the beneficiary population.

We studied factors affecting retention in the three HIV prevention and research scenarios described above among men enrolled in a high risk cohort of patients having current or past history of sexually transmitted infections (STI) in Pune, India. We explored demographic, behavioral and biological factors that might predict retention in the modeled scenarios of primary prevention programs, cohort studies and clinical trials.

## Methods

### Ethics Statement

The cohort studies were approved by the national and international scientific, ethics and regulatory committees or boards of National AIDS Research Institute, India and Johns Hopkins University, USA. All participants were enrolled after obtaining written informed consent as approved by the Ethics Committee.

Between 1993 and 2002, as part of collaborative studies between National AIDS Research Institute in Pune, India and Johns Hopkins University in the United States of America, cohort studies were undertaken in the industrial city of Pune located in the high HIV prevalence western state of Maharashtra in India. Using this dataset we carried out case-control analysis to study factors affecting retention of clients in HIV prevention research and programs. The “cases” in the three distinct modeled scenarios were selected from the cohort of male STI clinic attendees.

The overall aim of the parent cohort study was to prepare sites and generate baseline data for undertaking Phase I, II and III HIV prevention clinical trials. Men with current or past history of STI, female sex workers (FSWs) and non sex worker females (non-FSWs) attending STI clinics were enrolled in the parent cohort study after they received their HIV negative report. Thus all those who tested HIV negative were offered enrollment in a longitudinal study requiring quarterly visits for a period of two years as described in our previous papers [6], [7]. In this paper, we describe predictors of retention among men in the STI cohort using case-control analysis. Three modeled scenarios of Primary prevention, Cohort study and Clinical trials were identified as described previously.

### Participants

“Cases” represented individuals who were “retained” in the hypothetical scenarios created for the retention analysis of primary prevention, cohort studies and clinical trials described above. Age and time of recruitment matched “controls” were selected from the STI cohort in 1∶1 ratio.

### Defining outcome variable “retention” in three distinct modeled scenarios

1. *Retention in primary prevention scenario:* Individuals who returned for their first follow-up at 3 months after they received their HIV test report.
2. *Retention in cohort studies scenario:* Individuals who reported for follow-up to the study clinics at least once at the end of the first year and then at the end of the second year.
3. *Retention in clinical trials scenario:* Individuals who completed at least three scheduled visits both during the first year and the second year after enrolment.

In the parent cohort study from which this analysis is done, only standard counseling, offering HIV test and giving scheduled date for the next follow-up visit was done. No additional efforts were made to contact the participants either telephonically or through home visits to specifically improve retention.

### Statistical analysis

**Figure 1:**
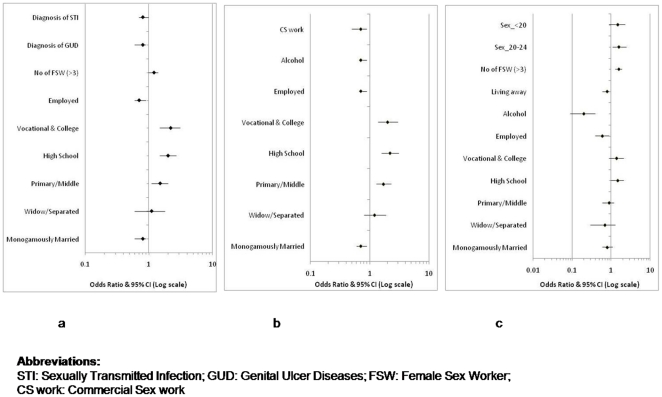
Predictors of retention in the modeled scenario of primary prevention (1a), cohort study (1b) and clinical trial (1c).

Univariate and multivariate conditional logistic regression analyses were performed to identify demographic (religion, marital status, education, employment), behavioral (living away from family, alcohol consumption, number of FSW partners, age at first sex, involvement in commercial sex work) and biological (tattooing, diagnosis of various types of STI, syndromic diagnosis of genital ulcer and discharge type of diseases) factors associated with retention in the three modeled scenarios respectively. The comparison of baseline characteristics of individuals in all the three scenarios was done using Chi-square or Fisher's Exact test whichever was applicable. The variables that were found to be significantly associated with retention in the univariate models were retained in the multivariate models. As an exception, the variable ‘number of FSW partners’ although not significant in the univariate model, was retained in the multivariate model due to its known relationship with retention [8], [9]. Forest plots in excel software were used to generate figures (1a – c) for multivariate analysis [10]. Data was analyzed using intercooled STATA version 10.0.

## Results

Between 1993 and 2002, a total of 14,137 individuals visited the STI clinics in this study. Of these, 10,801 (76%) were men, 3252 (23%) were women and 83 (0.5%) were eunuchs or trans-genders. Of all the 10, 801 screened male STI patients, 8631 (80%) were found to be HIV uninfected who were enrolled in the parent cohort study. The present case -control analysis is restricted to these enrolled men.

Most of the men were employed (89%), belonged to Hindu religion (81%), were living with their families (77%) and nearly 50% were ‘ever married’. More than half of these men reported history of alcohol consumption and 84% reported having FSW contact in the lifetime. The median age at initiation of sex among them was 19 years. Thirty two percent of the men presented themselves with the diagnosis of genital ulcer disease (GUD) (Data not shown in the tables).

### Profile of men in case control analysis in three modeled scenario

A total of 1286, 940 and 896 cases and equal number of matched controls were considered in three respective modeled scenarios of primary prevention, cohort study and clinical trials (Table 1). Cases and controls differed significantly for various baseline demographic and behavioral characteristics.

**Table 1:** Profile of men identified as cases and controls in three respective scenarios of primary prevention, cohort study and clinical trial.

Predictor variables	Primary Prevention Program			Cohort Studies			Clinical Trial		
	Cases = 1286n (%)	Controls = 1286n (%)	Chi-square or Fisher's exact *P*-value (two sided)	Cases = 940n (%)	Controls = 940n (%)	Chi-square or Fisher's exact *P*-value (two sided)	Cases = 896n (%)	Controls = 896n (%)	Chi-square or Fisher's exact *P*-value (two sided)
**Religion**Christian/PersianHinduMuslimBuddhist	32(2.5)1050(81.8)65(5.1)136(10.6)	37(2.9)1029(80.0)97(7.5)123(9.6)	0.06	26(2.8)752(80.0)56(6.0)106(11.3)	34(3.6)757(80.5)70(7.5)79(8.4)	0.09	22(2.5)741(82.8)41(4.6)91(10.2)	27(3.0)722(80.6)64(7.1)81(9.0)	0.09
**Marital Status**Never Married Monogamously MarriedWidow/ Divorced/ Separated	717(55.8)524(40.8)43(3.4)	644(50.1)607(47.2)35(2.7)	**0.004**	454(48.3)423(45.0)63(6.7)	393(41.8)490(52.1)57(6.06)	0.008	497(55.5)377(42.1)21(2.4)	450(50.2)415(46.3)31(3.5)	**0.05**
**Living away from family**NoYes	984(77.5)286(22.5)	950(76.5)292(23.5)	0.55	698(78.0)197(22.0)	639(74.3)221(25.7)	0.07	696(80.0)175(20.1)	656(75.4)214(24.6)	**0.02**
**Education**IlliteratePrimary/ MiddleHigh SchoolVocational and College	103(8.0)532(41.5)396(30.9)250(19.5)	179(14.0)598(46.6)334(26.0)172(13.4)	**<0.01**	122(13.0)401(42.8)259(27.6)156(16.6)	208(22.2)397(42.3)217(23.1)116(12.4)	**<0.001**	89(10.0)350(39.2)294(32.9)161(18.0)	111(12.5)446(50.1)214(24.0)120(13.5)	**<0.001**
**Employed**NoYes	199(15.5)1082(84.5)	128(10.0)1156(90.0)	**<0.01**	198(21.1)740(78. 9)	148(15.8)791(84.2)	**0.003**	145(16.3)747(83.7)	88(9.8)806(90.2)	**<0.001**
**Tattoo since last 2 years**NoYes	1106(94.4)66(5.6)	1100(94.7)62(5.3)	0.75	769(94.0)49(6.0)	765(93.1)57(6.9)	0.44	779(94.6)44(5.4)	762(94.0)49(6.0)	0.54
**Alcohol consumption**NoYes	49(39.5)75(60.5)	58(38.9)91(61.1)	0.92	875(93.1)65(6.91)	830(88.3)110(11.7)	**<0.001**	886(98.9)10(1.1)	832(92.9)64(7.1)	**<0.001**
**No of Female Sex Worker partners**Less than threeAbove 3	752(59.0)523(41.0)	797(62.4)481(37.6)	0.08	447(47.6)493(52.5)	451(48.0)489(52.0)	0.85	455(50.8)441(49.2)	544(60.7)352(39.3)	**<0.001**
**Age at first sexual contact**25 & above20–24Less than 20	100(7.8)464(36.4)711(55.8)	101(7.9)458(35.9)718(56.2)	0.96	66(7.0)306(32.6)568(60.4)	69(7.3)298(31.7)573(61.0)	0.91	52(5.5)314(35.0)530(59.2)	78(8.7)285(31.8)533(59.5)	**z0.04**
**Involved in Commercial Sex work**NoYes	1054(92.5)85(7.5)	1069(91.8)96(8.2)	0.48	771(92.6)62(7.4)	765(89.0)95(11.1)	**0.011**	684(92.1)59(7.9)	738(92.3)62(7.8)	0.89
**Diagnosis of Non Gonococcal Urethritis**NoYes	1115(95.8)49(4.2)	1093(96.1)45(4.0)	0.75	710(95.4)34(4.6)	606(95.4)29(4.6)	0.99	840(95.5)40(4.6)	735(95.5)35(4.6)	1.00
**Diagnosis of Granuloma Inguinale**NoYes	1265(99.5)07(0.6)	1262(99.6)05(0.4)	0.56	920(99.5)5(0.5)	907(99.7)03(0.3)	0.73	890(99.7)03(0.3)	875(99.1)08(0.9)	0.14
**Diagnosis of lymphogranulum venerum**NoYes	1244(97.8)28(2.2)	1228(96.9)39(3.1)	0.16	908(98.2)17(1.8)	893(98.0)18(2.0)	0.83	874(97.9)19(2.1)	859(97.4)23(2.6)	0.51
**Diagnosis of** **Genital warts**NoYes	1226(96.4)46(3.6)	1226(96.8)41(3.2)	0.59	894(96.7)31(3.3)	887(97.5)23(2.5)	0.30	862(96.5)31(3.5)	853(96.6)30(3.4)	0.93
**Diagnosis of GUD**NoYes	976(75. 9)310(24.1)	863(67.2)422(32.8)	**<0.01**	711(75.6)229(24.4)	698(74.4)240(25.6)	0.54	650(72.5)246(27.5)	613(68.6)281(31.4)	0.07
**Diagnosis of Genital Discharge**NoYes	1164(90.5)122(9.5)	1138(88.5)148(11.5)	0.09	829(88.2)111(11.8)	818(87.0)122(13.0)	0.44	814(90.9)82(9.1)	795(88.7)101(11.3)	0.14
**Diagnosis of STI**NoYes	774(60.2)512(39.8)	645(50.2)641(49.8)	**<0.01**	555(59.0)385(41.0)	538(57.2)402(42.8)	0.43	506(56.5)390(43.5)	460(51.3)436(48.7)	**0.03**
**Diagnosis of Non GUD STI**NoYes	1046(81.3)240(18.7)	1013(78.8)273(21.2)	0.10	784(83.4)156(16.6)	778(82.8)162(17.2)	0.71	726(81.0)170(19.0)	704(78.6)192(21.4)	0.11

CI: Confidence Interval.

### Predictors of retention in scenario 1: primary prevention

Marital status, education, employment, diagnosis of GUD and diagnosis of any STI were found to be associated with retention in the univariate analysis (Table 2). In the multivariate analysis (Fig. 1a), men who were married and monogamous (*p* = 0.03), employed (*p* = 0.02) and those with the clinical diagnosis of GUD (*p* = 0.04) were less likely to return for the follow up visit. In contrast, male STI patients reporting higher level of education (*p*<0.001) and those who had more than three FSW partners were more likely to report back for follow-up *(p = 0.03)*.

**Table 2:** Univariate analysis of Predictors of Retention in three modeled scenarios.

Predictor variables	Primary Prevention Program			Cohort Studies			Clinical Trial		
	Odds ratio	95% CI	*P*-value(two sided)	Odds ratio	95% CI	*P*-value(two sided)	Odds ratio	95% CI	*P*-value(two sided)
**Religion**Christian/PersianHinduMuslimBuddhist	Referent1.20.81.3	0.7,1.90.4,1.40.7,2.8	0.500.380.37	Referent1.31.01.8	0.8,2.20.6,1.90.9,3.2	0.330.890.06	Referent1.30.81.4	0.7, 2.30.4,1.60.8,2.6	0.370.530.28
**Marital Status**Never Married Monogamously MarriedWidow/ Divorced/ Separated	Referent0.70.9	0.5,0.80.5,1.5	**<0.001**0.66	Referent0.70.8	0.5, 0.80.6,1.3	**<0.001**0.38	Referent0.70.5	0.5,0.90.3,0.9	**0.004** **0.024**
**Living away from family**NoYes	Referent0.9	0.8,1.1	0.53	Referent0.8	0.7,1.0	0.07	Referent0.8	0.6,0.9	0.025
**Education**IlliteratePrimary/ MiddleHigh SchoolVocational and College	Referent1.62.22.7	1.2,2.11.6,2.91.9,3.7	**0.001** **<0.001** **<0.001**	Referent1.82.12.4	1.4, 2.31.6, 2.91.7, 3.4	**<0.001** **<0.001** **<0.001**	Referent1.01.81.8	0.7,1.41.3,2.51.2,2.6	0.960**0.001** **0.003**
**Employed**NoYes	Referent0.6	0.5,0.7	**<0.001**	Referent0.7	0.5, 0.9	**0.003**	Referent0.5	0.4, 0.7	**<0.001**
**Tattoo since last 2 years**NoYes	Referent1.1	0.7,1.5	0.78	Referent0.9	0.6, 1.3	0.43	Referent0.9	0.6,1.4	0.60
**Alcohol consumption**NoYes	Referent1.0	0.6,1.7	0.92	Referent0.6	0.4,0.8	**<0.001**	Referent0.2	0.08,0.3	**<0.001**
**No of Female Sex Worker partners**Less than threeAbove 3	Referent1.2	0.9,1.4	0.08	Referent1.0	0.9,1.2	0.85	Referent1.5	1.2,1.8	**<0.001**
**Age at first sexual contact**25 & above20–24Less than 20	Referent1.01.0	0.7,1.40.7,1.4	0.900.97	Referent1.11.0	0.7,1.60.7,1.5	0.710.85	Referent1.71.5	1.2,2.61.0,2.3	**0.008** **0.03**
**Involved in Commercial Sex work**NoYes	Referent0.9	0.7,1.2	0.53	Referent0.7	0.5,0.9	**0.01**	Referent1.0	0.7,1.5	0.923
**Diagnosis of Non Gonococcal Urethritis**NoYes	Reference1.1	0.7,1.6	0.71	Referent1.0	0.6,1.7	0.88	Referent0.9	0.6,1.6	0.99
**Diagnosis of Granuloma Inguinale**NoYes	Reference1.4	0.4,4.4	0.57	Referent1.6	0.4,6.9	0.50	Referent0.4	0.1,1.4	0.14
**Diagnosis of lymphogranulum venerum**NoYes	Reference0.7	0.4,1.2	0.16	Referent0.9	0.5,1.8	0.82	Referent0.8	0.4,1.5	0.50
**Diagnosis of Genital warts**NoYes	Reference1.1	0.7,1.7	0.60	Referent1.4	0.8,2.3	0.29	Referent1.0	0.6,1.7	0.91
**Diagnosis of GUD**NoYes	Referent0.6	0.5,0.8	**<0.001**	Referent0.9	0.8,1.2	0.54	Referent0.8	0.7,1.0	0.07
**Diagnosis of Genital Discharge**NoYes	Referent0.8	0.6,1.0	0.09	Referent0.9	0.7,1.2	0.44	Referent0.8	0.6,1.1	0.14
**Diagnosis of STI**NoYes	Referent0.7	0.6,0.8	**<0.001**	Referent0.9	0.8,1.1	0.43	Referent0.8	0.7,0.9	**0.03**
**Diagnosis of Non GUD STI**NoYes	Referent0.9	0.7,1.0	0.10	Referent0.9	0.8,1.2	0.71	Referent0.9	0.7,1.1	0.19

### Predictors of retention in scenario 2: cohort study

In the univariate analysis, marital status, education, employment, alcohol consumption and involvement in sex work were observed to be associated with retention (Table 1). In the multivariate analysis (Fig. 1 b), men who were married monogamous *(p = 0.001)*, employed *(p = 0.001)*, who gave history of alcohol consumption *(p = 0.002)* or those who were involved in sex work *(p = 0.001)* were 30% less likely to be retained in the cohort study. All these variables were found to be independent predictors of lower retention. However, men who were educated to high school and beyond were almost 2 times more likely to be retained in the cohort study scenario *(p<0.001)*.

### Predictors of retention in scenario 3: clinical trials

Marital status, living away from the family, education, employment, alcohol consumption, number of FSW partners, age at first sexual intercourse and diagnosis of STI were significantly associated with retention in the clinical trial scenario in the univariate analysis (Table 1). In the multivariate analysis, independent predictors of retention were living away from the family (*p* = 0.04), being employed (*p* = 0.003) and habit of alcohol consumption (*p*<0.001). More educated male patients or those who had more than three FSW partners or those who initiated sex at an older age were almost 1.5 times more likely to be retained and maintain rigorous follow-up schedule of a clinical trial scenario (Fig. 1c).

## Discussion

We have used data from large cohort studies on STI patients in Pune, India in modeled scenarios to study the extent of retention and determinants of retention in male STI patients that constitutes an important bridge population in HIV transmission in India [11]. We have identified demographic, behavioral and biological factors that might predict adherence/ non adherence of male STI patients to suggested visit schedules. We expect that this knowledge would be very useful to design specific strategies that might assist in optimizing retention in HIV prevention research and programs. It is possible to identify potential defaulters for retention and implement appropriate interventions. This might be less expensive than tracking patients or research participants after enrollment.

Being employed was a common predictor of lower retention across all the three study models. Level of education showed likelihood of retention across all three modeled scenarios. Education level among high risk men in India is low [12]–[13]. Additional efforts are required to be made for the less educated or illiterate men to effectively retain them in primary prevention programs and clinical trials. Similar observations have been made in other studies among men who have sex with men [14]–[16]. Our observation also corroborated with a similar observation in NIMH HIV prevention trial [17]. As majority of VCT center attendees in the Government sector facilities in India are less educated [18], special efforts to improve their retention in primary prevention will be required. Additionally, we observed that retention was less among employed men although the education level is expected to be high among them. Paucity of time could be the logical limiting reason for employed men to come for repeated follow-up visits as reported by several investigators [19]–[21]. To facilitate retention, it might be necessary to keep the health facilities and research clinics open and available out of routine work hours.

Presence of GUD, history of commercial sex work and living away from the family were predictors of lower retention in primary prevention, cohort study and clinical trial models respectively. Alcohol consumption predicted lower retention in the cohort study and clinical trial models while the married monogamous men had lower likelihood of retention in the primary prevention and cohort study models.

It is well known that in therapeutic programs, benefits are generally immediate and more readily visible. In contrast, success of prevention programs lies in better, sustained and prolonged utilization of services which indicates ‘retention needs’. Retention in primary prevention and allied research is expected to be dependent on many factors and strategies such as retention counseling, quality of delivery of programmatic and research activities, and participant related factors such as motivation, costs and time required to be spent by them. As the prevention programs mature and new prevention trials are undertaken, the need to identify potential drop outs has to be addressed on priority. Optimizing retention of the end-users is crucial for assessing efficacy [22] and hence strategies should be considered to address various factors influencing retention during implementation of prevention programs and research. Predictors of retention identified in the study could be used for developing an instrument to identify the clients who are likely to fail to return for required follow-up visits either in prevention program or in prevention research. Using such an instrument could be a cost effective strategy to minimize ‘drop-outs’ rather than using expensive measures to track participants or patients who are lost to follow-up later.

It has been suggested that both prevention and adherence science need to expand beyond individual boundaries to learn more about motivational and structural strategies that can be applied to large populations so that prevention technologies have adequate time to prove useful when implemented in the communities [5]. Therefore it is relevant to explore individual factors as well as those related to individual's family or societal environment that can prevent retention in prevention or research programs.

Poor sexual health seeking behavior among men despite their high risk behavior poses a grave challenge [23]. We observed that married men, who were monogamous, were less likely to be retained in prevention programs and cohort study scenarios in this study. The precise reasons for this observation may have to be explored through qualitative studies. Important role of spouses in men's health seeking has been reported [24]. Several studies have also reported that men who are living away from spouse as well as divorced or single individuals have high risk behaviors [12] and higher dropout rate from the offered prevention umbrella [25]–[28]. Our observation that men who were ‘living away from family’ were less likely to be retained in the clinical trials scenario provides supporting evidence to this possibility. All these observations are strongly suggestive of better health seeking by men having family support. We feel that couple centered approach and involvement of female partners in male oriented programs may contribute to the success of program for men. However, this approach has an inherent limitation that men will have to share information about their health and sickness with their spouses. Counseling sessions in programs and research could focus on specifically discussing the role of spouses and families not only in improving health seeking, but also in keeping up with the visit schedules of programs or studies they are participating in.

Among the behavioral characteristics, those men who reported having more than three female sex worker partners were more likely to return for follow-up in the primary prevention and clinical trial scenarios. This probably reflects men's ‘self perception’ about their risk behavior. Health seeking in terms of regular and frequent follow up is perhaps better among men practicing high risk behavior. Focused attention would be required to be given on men reporting high risk behavior less frequently. There is an opportunity to effectively intervene to achieve behavioral change through prevention programs.

In India, male commercial sex work is all but invisible and not much is currently known about the status of male sex workers although some studies have reported high HIV prevalence among them indicating a need to develop new [29], innovative interventions targeted towards men in commercial sex work. In the present study among male STI patients, men reporting commercial sex work were less likely to be retained in the cohort study scenario. This is a high risk population and a reliable estimate of HIV incidence in this category of men is an important public health need. Additionally this population would also be targeted for Phase IIb or III studies of HIV prevention technologies and their retention in future clinical trials would be very critical. Lower age at sex initiation has been reported to be associated with early HIV infection in this cohort [30]. Hence, emphasis should be given on targeting younger men in prevention programs and ensuring their continued retention in the programs to sustain safer behavior. Alcohol intake has been reported as a predictor of non-retention in several studies [17], [31], [32]. It was no surprise to find that men who gave a history of alcohol consumption were less likely to be retained in our study as well. Long term commitment might be a challenge in cases of alcohol addiction. It might be important to emphasize on identification of alcohol consuming behavior at the entry point of prevention settings and making special efforts to ensure retention of alcohol consuming individuals under the HIV prevention umbrella.

The diagnosis of GUD was an independent predictor of return for a follow-up visit within 3 months of enrollment i.e. primary prevention scenario. This observation has specific public health significance because it provides opportunities and complete treatment of GUD and appropriate counseling for behavior change. We have already reported decline in HIV acquisition risk with decline in GUDs [7]. GUDs are “visible or noticeable” STI that could motivate a person to seek further medical advice and hence such individuals are probably more likely to return to the study clinics. However, it has been reported that non-GUD STIs are also associated with high HIV prevalence [33]–[34]. Hence, it is advisable that men with clinically invisible or non-apparent STIs should also be targeted for HIV prevention interventions and retention counseling. Interactive counseling approaches directed at a patient's personal risk, the situations in which such a risk is likely to occur and the use of goal-setting strategies are effective in STI/ HIV prevention [35]. Shepherd et al [36] have provided evidence that by enhancing access to treatment and interventions through mechanisms such as counseling, education, and provision of condoms for prevention of STIs, especially GUD among disadvantaged men, the disparity in rates of HIV incidence could be lessened considerably. As part of the clinical interview, health-care providers should routinely and regularly obtain sexual histories from their patients and plan retention management measures along with implementing measures for risk reduction. It is important to ensure that the clients continue to practice safe behavior through sustained follow-up.

We recommend that counselors working with participants and beneficiaries of research studies and program should specifically take into consideration clients' occupation, current marital relationship, habit of alcohol consumption, possibility of non-GUD STI, and identify cases that may have a potential for being lost to follow-up. This strategy may prove to be cost effective, less cumbersome and easier to ensure high retention. In future, the identified predictors in this study could be used to develop a counseling check-list with measurable indicators of failure in retention. Such a tool would require validation studies in prevention programs and clinical trial settings.

The recruitment of participants in this study was through public sector based STI clinics which is a limitation for generalizability of the findings. The profiles of clients visiting the public and private sector facilities available are known to be different [37]–[38]. Since VCT was primarily offered in a research context in this study, lessons learnt may have some limitations in terms of applicability to primary prevention programs rolled out to masses. Hence the predictors of retention identified in this study will have to be understood appropriately in context of the patients receiving health care in other facilities. Secondly, the study essentially involves men and in India men are not only the key decision makers in the community and families but also the major contributors to transmission of HIV in India [20], [39]. The National Family Health Survey III data [40] in India has shown that 10–15% of Indian men are at risk of HIV infection. Hence studies to identify predictors of retention among men gains significance. However, the predictors of retention among women are likely to be different and they must be explored.

We conclude that achieving high levels of retention and preventing drop outs was a challenge in case of all the three scenarios of primary prevention, cohort studies and clinical trials. The knowledge about identified predictors of sub-optimal retention could be useful in developing appropriate retention checklists or tools in case of the above-mentioned prevention and research programs to minimize potential drop-outs.
